# Propolis - based chitosan varnish: drug delivery, controlled release and antimicrobial activity against oral pathogen bacteria

**DOI:** 10.1186/1472-6882-14-478

**Published:** 2014-12-12

**Authors:** Juçara R Franca, Mariana P De Luca, Tatiana G Ribeiro, Rachel O Castilho, Allyson N Moreira, Vagner R Santos, André AG Faraco

**Affiliations:** Pharmaceutical Science Post-Graduation Program, Faculty of Pharmacy, Federal University of Minas Gerais, Belo Horizonte, Minas Gerais Brazil; Department of Restorative Dentistry, Faculty of Dentistry, Federal University of Minas Gerais, Belo Horizonte, Minas Gerais Brazil; Department of Oral Clinical, Oral Pathology and Oral Surgery, Federal University of Minas Gerais, Belo Horizonte, Minas Gerais Brazil; Faculdade de Odontologia UFMG, Campus Pampulha, Av. Pres. Antonio Carlos, 6627, Pampulha, Belo Horizonte, MG 31270-901 Brasil

**Keywords:** Propolis, Chitosan, Varnish, Drug delivery, Cariogenic biofilm, Prevention, Oral microorganisms

## Abstract

**Background:**

Dental caries is the most prevalent oral disease in several Asian and Latin American countries. It is an infectious disease and different types of bacteria are involved in the process. Synthetic antimicrobials are used against this disease; however, many of these substances cause unwarranted undesirable effects like vomiting, diarrhea and tooth staining. Propolis, a resinous substance collected by honeybees, has been used to control the oral microbiota. So, the objective of this study was to develop and characterize sustained-release propolis-based chitosan varnish useful on dental cariogenic biofilm prevention, besides the *in vitro* antimicrobial activity.

**Methods:**

Three formulations of propolis - based chitosan varnish (PCV) containing different concentrations (5%, 10% and 15%) were produced by dissolution of propolis with chitosan on hydro-alcoholic vehicle. Bovine teeth were used for testing adhesion of coatings and to observe the controlled release of propolis associated with varnish. It was characterized by infrared spectroscopy, scanning electron microscopy, casting time, diffusion test *in vitro* antimicrobial activity and controlled release. Minimum inhibitory concentration (MIC) and minimum bactericidal concentration (MBC) were tested for the main microorganisms involved in the cariogenic biofilm through the microdilution test in 96-well plates.

**Results:**

The formulations presented a tooth surface adherence and were able to form films very fast on bovine tooth surface. Also, propolis-based chitosan varnishes have shown antimicrobial activity similar to or better than chlorhexidine varnish against all oral pathogen bacteria. All microorganisms were sensitive to propolis varnish and chitosan. MIC and MBC for microorganisms of cariogenic biofilme showed better results than chlorhexidine. Propolis active components were released for more than one week.

**Conclusion:**

All developed formulations turn them, 5%, 10% and 15% propolis content varnish, into products suitable for clinical application on dental caries prevention field, deserving clinical studies to confirm its *in vivo* activity.

## Background

Dental caries is the most prevalent oral disease in several Asian and Latin American countries, despite the significant decline in many developed countries over recent decades [1–4]. Moreover, it is expected to increase in many developing countries in Africa [5–7]. Although its incidence is particularly high during childhood [3, 8–10], people are susceptible throughout their lifetime [11, 12]. Dental caries is an infectious disease, the development of which is a dynamic process involving alternating demineralization and remineralization, rather than unidirectional demineralization [13]. Demineralization of tooth structure is caused by the organic acids produced in dental plaque biofilm by the metabolic action of the cariogenic microorganisms on fermentable carbohydrates [14, 15]. Formation of biofilm is a biological process associated with the attachment, detachment and proliferation of oral bacteria on the tooth surfaces. The dental biofilm is formed via adhesion of planktonic bacteria to a protein pellicle coating the tooth surfaces [16, 17]. Many types of bacteria participate in the formation of the dental biofilm [18]. Dental caries is caused by the interaction, over the time, of bacteria that produce acids with a substrate that the bacteria can metabolize and many host factors that include teeth and saliva [19]. There are only a few groups of microorganisms that can adhere to the teeth, which includes mutans *Strepcococci* group, *Lactobacillus* and some *Actinomyces* species [20, 21]. More than five *Streptococcus* species and *Actinomyces viscosus* are regarded as early colonizers of tooth surfaces, while mutans *Streptococci* such as *S. sobrinus, S. salivarius, S. sanguinis* and *S. mutans* are considered middle important colonizers of the dental biofilm [19]. The inhibition of plaque biofilm formation is the key to successful control and prevention of dental caries. Previous antibacterial mouth rinses, which generally contain fluorides, alcohols, detergents and other antimicrobial substances, effectively reduce plaque formation. Synthetic antimicrobials used in tooth pastes and mouth rinses include povidone iodine products, chlorhexidine, cetylpyridinium chloride, triclosan and zinc citrate [22, 23]. However, many of these substances cause unwarranted undesirable effects like vomiting, diarrhea and tooth staining. Prevention, based on common risk factors, plays an important role on caries management [24, 25] and recent studies have shown the anti-caries activity of different natural products such as propolis [26, 27].

Propolis is a resinous substance collected by honeybees from buds and exudates of certain trees and plants and stored inside their hives. It has been used in folk medicine from ancient times to treat various ailments [28]. Propolis has been used for centuries by world population due to its pharmacological properties such as anti-inflammatory, healing, antimicrobial and antioxidant [29]. In dentistry, propolis has been used to control the oral microbiota [30]. The antibacterial activity of propolis is reported due to flavonoids, aromatic acids, and esters present in resins. Galangin, pinocembrin, and pinostrobin are known as the most effective flavonoids agents against bacteria. Ferulic acid and caffeic acid also contribute to the bactericidal action of propolis [31]. Propolis has plenty of biological and pharmacological properties and its mechanisms of action have been widely investigated in the last years, using different experimental models *in vitro* and *in vivo*[26].

Another potential agent on caries prevention is chitosan, which is a polysaccharide comprising copolymers of glucosamine and N-acetyl- glucosamine [32]. Water-soluble chitosan have proven to be active against Prevotella gingivalis (MIC = 0.31 mg/mL) and Fusobacterium nucleatum (0.08 mg/mL) [33]. In the same way, high-molecular weight and low molecular chitosan showed activity against a sort of different anaerobic oral bacteria like P. gingivalis (MIC = 1 and 1 mg/mL respectively), T. forsythensis (MIC = 1 and 1 mg/mL respectively), P. buccae (MIC = 3 and 1 mg/mL respectively), A. actinomycetemcomitans (MIC = 5 and 3 mg/mL respectively), P. intermedia (MIC = 1 and 3 mg/mL respectively) and S. mutans (MIC = 3 and 5 mg/mL respectively) [34]. Adhesion of low molecular weight chitosan formulations on tooth analogs was demonstrated [35, 36]. Chitosan, also, offers a flexible platform for designing coatings to protect surfaces from infection, since it was been demonstrated that chitosan-coated surfaces resists to biofilm of *Staphylococcus epidermidis*, *Staphylococcus aureus*, *Klebsiella pneumoniae*, *Pseudomonas aeruginosa* and *Candida albicans*[37]. Varnishes are pharmaceutical formulations that can form antimicrobial tooth film and protect the tooth surface of caries [38]. Here, we report the development, the characterization and antimicrobial activity of sustained release chitosan-based propolis varnishes,for possible use in the prevention of cariogenic biofilm.

## Methods

### Propolis samples

Green propolis used in this study was collected during spring, and obtained from a commercial beekeeping named Pharmanéctar® registered at the National Agency of Sanitary Surveillance (ANVISA register n° 17.226.994/0001-61), in Minas Gerais State, Brazil. Botanical origin of Brazilian green propolis was *Baccharis dracunlifolia*[29, 39]. Ethanolic propolis extract (EPE) was used in this study and the chemical composition is shown in Table 1. Crude propolis samples were further dehydrated with a low-vacuum pump, and it was prepared as described [40]. The dried propolis samples were ground into fine powder, and 2.5 g of propolis was mixed with 25 mL of 80% aqueous ethanol in a test tube and shaken at 70°C for 30 min. After extraction, the mixture was centrifuged at 8000 × g to obtain the supernatants, which were named alcoholic propolis extract, which was used in this study.

**Table 1:** Chemical composition* of the green Propolis extract

Component	Quantity (mg/g)	Component	Quantity (mg/g)
Coumaric acid	3.56	Pinobanksin-3-acetate	13.92
Cinamic acid	1.66		
Quercetin	1.38	Crisin	3.51
Kaempferol	1.77	Galangine	9.75
Isoramnetin	0.91	Kaempferide	11.60
Sakuranetin	5.57	Artepillin-C	82.96

*PharmaNectar, Brazil, 2007.

### Chemicals

Chitosan of medium molecular weight (75-85% of desacetylation) was purchase from Sigma-Aldrich (Michigan, USA). Ethanol was purchased from Synth (Sao Paulo, Brazil) and glacial acetic acid (100%) was purchased from Merck (USA). Purified water was obtained from a Milli-Q water purification system. All reagents used were analytical grade. Brain Heart Infusion (BHI) broth, BHI agar, rogosa broth, rogosa agar menadione and vitamin K was purchased from Difco (USA). Chlorhexidine 0.12% varnish was purchased from Sigma (USA).

### Varnishes preparation

Propolis-based chitosan varnishes (PCV) were prepared by the addition of acetic acid to EPE 25%. After mixing, medium molecular weight chitosan was added and water was used to complete the formulation volume. Then, the compositions were mixed overnight and the varnishes formulations containing 5% w/v, 10% w/v and 15% w/v of propolis (PCV 5%, PCV 10% and PCV 15%, respectively) were developed and characterized. The chitosan base varnish (CHV), used as blank varnish, were prepared by the same method, using ethanol instead of EPE. Varnish compositions are presented in Table 2. Final pH of the formulation was between 4.0 and 5.0.

**Table 2:** **Composition of the Propolis varnishes containing 15 w/v % (PCV 15%), 10 w/v % (PCV 10%) e 5% w/v % (PCV 5%) of Propolis extract and of the blank varnish (BCV)**

Component	PCV 5%	PCV 10%	PCV 15%	BCV
Propolis ethanolic extract (25%)	20.0 mL	40.0 mL	60.0 mL	-
Ethanol	40.0 mL	20.0 mL	-	60.0 mL
Acetic acid	9.0 mL	9.0 mL	9.0 mL	9.0 mL
Chitosan	1.0 g	1.0 g	1.0 g	1.0 g
MilliQ water q.s.p.	100 mL	100 mL	100 mL	100 mL

### Attenuated total reflection fourier transform infrared spectroscopic imaging (ATR-FTIR) analyses

Formulations, PCV, CHV and EPE were dried for 30 minutes in Petri dishes before ATR-FTIR analysis. The spectra of the dried samples were recorded within films on a PerkinElmer FTIR spectrometer, model Spectrum One.

### Varnish casting time

Varnishes casting time was determined by applying 50 μL directly onto the buccal surface of incisive bovine teeth using a sterile swab. Bovine teeth were donated by specialized slaughterhouse authorized by the city of Belo Horizonte in July 2010. The teeth were collected from carcasses that would be incinerated, and the animals were not the direct object of study and therefore not submitted to the Ethics Committee on Animal Research. After the application, the time needed to form a completely casted film on tooth surface was determined. The casting time was determined using or not compressed air as casting agent. The test was performed in triplicate in four teeth for each formulation.

### Scanning electron microscopy (SEM) analysis

The morphology of the formed films on the tooth surface was studied using a JEOL scanning electron microscope, model JSM-6360LV, operating in 15 kV acceleration voltage. The samples were prepared by the application of 50 μL of the varnishes in incisive cow tooth using a swab. After the formation of the completely casted films, the teeth were broken and directly analyzed at suitable acceleration voltages for each sample, in different magnification ranges. Representative micrographs were taken.

### *In vitro*antimicrobial susceptibility test

The antimicrobial susceptibility test was made according to the agar diffusion method [41]. Microorganisms *S. mutans* (ATCC 25175), *S. sanguinis* (ATCC 10557)*, S. salivarius* (INCQS 00457), *L. casei* (ATCC 393), *Aggregatibacter actinomycetemcomitans* (ATCC 33384), *Porphyromonas gingivalis* (ATCC 33277)*, Prevotella intermedia* (ATCC 25611), and *Fusobacterium nucleatum* (ATCC 23726) were used in the experiment. *Streptococci* group was cultivated in BHI broth and after 18 hours, was transferred into BHI agar containing 5% sucrose. *Lactobacillus casei* was suspended in Rogosa broth, and after 18 hours of growth were transferred to plates containing Rogosa agar. The other anaerobic microorganisms were suspended in BHI broth supplemented with sheep blood and after 24 hours were transferred to petri dishes containing BHI agar supplemented with sheep blood, 1% menadione and 1% vitamin K. The inocula of all microorganisms were prepared by collecting 3–5 colonies grown on agar after 24 hours and the number of microorganism was calculated based on the standard turbidity of 0.5 McFarland, corresponding to 1.0 × 10^8^ colony forming units (CFU)/mL. *Lactobacillus* and *Streptococcus* were grown at 37°C in atmosphere containing 5% CO_2_, while other microorganisms were cultured anaerobically at 37°C. Sterile paper blank discs containing 50 μL of each propolis varnish were placed on the agar. CHV were used as negative control, chlorhexidine 0.12% varnish (CHL) and EPE 5% were used as inhibition growth positive controls. The inhibition of bacterial growth was determined by measuring the diameter of inhibition zones formed after 48 hours of incubation. The solvent effect was annulated by casting the discs containing the samples (PCV, CHV, CHL and EPE) before placing them in the agar. MIC and MBC planktonic tests were made according to the rules of Clinical and Laboratory Standards Institute (CLSI, 2007) [41].

### Drug release

Propolis release profile from formulations was studied in 3 mL of medium, which was a solution containing 20% v/v of ethanol in water, at 37°C. The samples were prepared by the application of 40 μL (20 μL twice) of the varnishes in incisive bovine teeth, previously cut in 0.5 × 0.5 cm square pieces. After the formation of the completely casted films, the teeth were placed in tubes with the same medium used before (3 mL of 20% v/v of ethanol in water). At scheduled time intervals (30 min, each hour until eight hours, 24 h, and then, once a week until the end of the release), samples were taken and the entire medium volume (3 mL) was replaced with fresh medium to maintain sink conditions. The amount of propolis released was quantified as total flavonoids content, measured as quercetin) [42]. UV/Vis spectroscopy at 425 nm was used for flavonoids quantification and the method was properly validated, according to ICH guidelines before analysis (linear equation: y = 0.106722 × +0.0229511, r^2^ = 0.9970, n = 5).

### Statistical analysis

The statistical analyses were performed using GraphPad PrismTM (version 5.0 for Windows). The data are representative of three different experiments, which presented similar results. UV-visible spectrophotometric assay was determined by linear regression and the r^2^ value indicated the measure of goodness-of-fit for linear regression. The Pearson normality test was used to determine the normality of the data. Since the data were found to be normally distributed, the Unpaired Student’s t-test was used.

## Results

Propolis and chitosan formed a completed mixed formulation and no interfaces separation were find between varnish components.

### ATR-FTIR analysis

**Figure 1:**
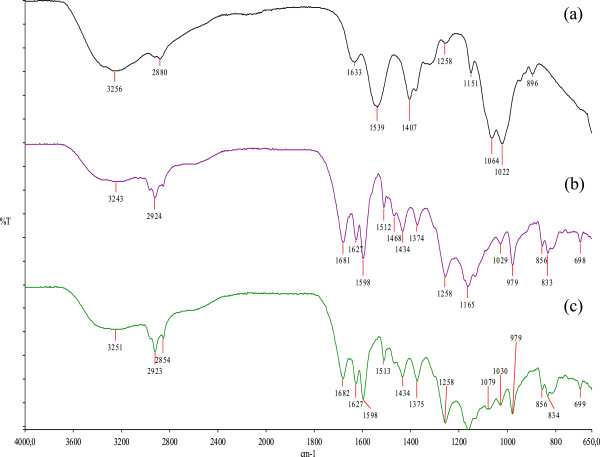
**ATR-FTIR spectrum of a, blank varnish (CHV); b, Ethanolic Propolis extract (EPE) and c, Propolis varnish containing 15% of dried extract (PCV 15%).**

ATR-FTIR spectrum of CHV (a), EPE (b) and PCV 15% (c), after casting, are presented in Figure 1. In CHV spectrum, it is possible to identify characteristic absorption bands of chitosan: an overlapped wide absorption band at 3256 cm^-1^ due to the stretching vibration of the O-H and N-H bonds; two bands at 1633 and 1539 cm^-1^, which can be attributed to amide I (C = O stretching) and to N-H (amine) vibration overlapped to amide II (N-H vibration), respectively and, at 1064 and 1022 cm^-1^, two bands that can be attributed to C-O-C and C-O vibration. From propolis extract, typical bands at 1165 cm^-1^, attributed to C–O and C–OH vibration, at 1434 cm^-1^, attributed to C–H vibration, at 1512, 1598 and 1630 cm^-1^, attributed to aromatic ring deformations and at 1681 cm^-1^, attributed to C = O stretching of flavonoids and lipids, found in propolis ethanolic extract. The spectrum of PCV showed characteristic bands both of CHV (e.g. 1079 cm^-1^) and EPE (e.g. 1681, 1627, 1598, 1513 and 1434 cm^-1^), with slight and no significant shifts. Moreover, the first band of ATR-FTIR spectra was widened and stiffened to a higher frequency compared to PCV spectrum (from 3243 to 3251 cm^-1^), suggesting the hydrogen bonding between chitosan and propolis. There are no new bands in PCV spectrum. The ATR-FTIR spectra of PCV 10% and 5% showed the same pattern of PCV 15% (data not shown).

### Varnish casting time

The casting times of the formulations on tooth surface without using compressed air for CHV, PCV 15%, PCV 10% and PCV 5% were about thirty minutes (Table 3). Although the vehicle of the varnishes does not casts fast naturally, the casting process is accelerated by the compressed air, which gives a casting time of less than five minutes for all the formulations (Table 3). Propolis alcoholic extract ran of the teeth and was not able to form film on tooth surface. The films which were formed on the teeth surface kept their integrity even after 24 hours of immersion and shacking, but were easily removed by brushing. No signal of change on tooth color surface was observed after brushing.

**Table 3:** Casting times (in minutes) of Propolis varnishes containing 15%, 10% and 5% of Propolis (PCV 15%, PCV 10% and PCV 5%, respectively) and blank varnish (BCV), after application of 50 μL of the formulation on incisive tooth surface*

Formulation	PCV 15%	PCV 10%	PCV 5%	BCV
Natural casting time	44.50 ± 0.35^a^	40.84 ± 1.03^a^	37.20 ± 2.67^a^	31.46 ± 0.22^a^
Compressed air helped casting time	5.63 ± 0.18^b^	4.32 ± 0.17^b^	3.89 ± 0.18^b^	2.62 ± 0.40^b^

*Different letters in the same columns means statistically significant different values.

Media and Standard Deviation (M ± SD) of three experiments. Unpaired Student’s t-test. p values less than 0.05 were considered significant.

### SEM analysis

**Figure 2:**
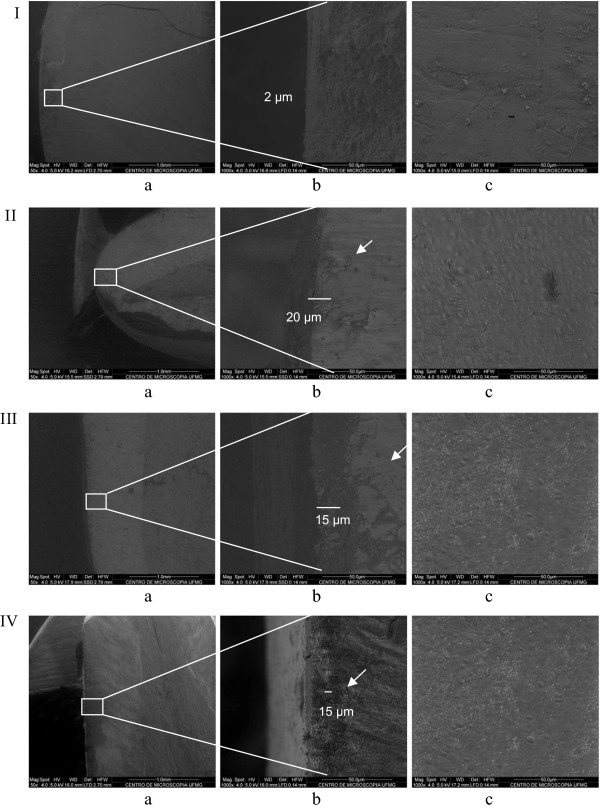
**Morphological characterization of films which was formed on tooth surface after application of Chitosan-based Propolis varnishes.** SEM pictures of I: blank Chitosan varnish (BCV); II: Propolis varnishes containing 15% of Propolis extract (PCV 15%); III: Propolis varnishes containing 10% of Propolis extract (PCV 10%); and IV: Propolis varnishes containing 5% of Propolis extract (PCV 5%); **a**, side view; **b**, expansion of a; **c**, surface view.

SEM pictures of CHV, PCV 15%, PCV 10%, and PCV 5% are presented on Figure 2. The arrows indicate the film formed by the varnish. Thickness is also indicated. From surface pictures (Figure 2c), it was observed that the film formed was uniform and homogeneous and lateral pictures showed that the film was compact and had between 15 and 20 μm of thickness. The films were in intimate contact with the tooth surface and no sign of possible detachment between the film and the tooth surface was indentified.

### Antimicrobial susceptibility test

All concentrations of propolis-chitosan varnish inhibited the growth of all microorganisms tested (Table 4). However, no significant difference was observed between the varnishes of 5%, 10% and 15%. On the other hand, the zones of inhibition were lower for anaerobic microorganisms *P. gingivalis* and *A. actinomycetemcomitans*, while larger zones of inhibition were observed for *P. intermedia* and *F. nucleatum* . No significant difference was observed between the zones of inhibition of the cariogenic biofilm microorganisms *S. mutans*, *S. sanguinis*, *S. salivarius* and *L. casei. C. dubliniensis* responded better the varnish inhibition when compared *C. albicans* and *C. lusitaniae* . Both varnishes, as well as the EPE 5% (control) exhibited higher inhibition zones for most microorganisms tested when compared with controls CHV, NYS and CHL . The minimum inhibitory concentration (MIC) and minimum bactericidal concentration (MBC) for cariogenic microorganisms (Table 5) were lower compared with controls EPE, CHV and CHL.

**Table 4:** **Susceptibility test of propolis- based chitosan varnish against oral pathogens microorganisms**

Microorganism	Inhibition zones (M ± SD) = mm						
	PCV			CHV	EPE	NYS	CHL
	5%	10%	15%				
*S. mutans*	20.3 ± 0.51	20.2 ± 0.88	21.0 ± 0.00	10.4 ± 0.22	22.6 ± 0.66	-	19.0 ± 0.00
*S. sanguinis*	21.5 ± 0.25	21.5 ± 0.25	22.3 ± 1.18	10.5 ± 0.25	21.3 ± 0.31	-	19.0 ± 0.00
*S. salivarius*	20.5 ± 0.33	20.5 ± 0.33	21.5 ± 0.50	8.30 ± 0.33	20.5 ± 0,55	-	18.5 ± 0,55
*L. casei*	19.3 ± 0.25	19.3 ± 0.25	21.3 ± 0.71	9.50 ± 0.55	16.0 ± 0.00	-	17.3 ± 0.33
*P. intermedia*	26.3 ± 1.03	23.3 ± 1.13	24.0 ± 0.00	10.6 ± 0.22	17.0 ± 0.00	-	20.3 ± 0.53
*P. gingivalis*	13.0 ± 0.00	11.6 ± 0,23	8.30 ± 0.33	11.5 ± 0.85	14.3 ± 1.13	-	13.3 ± 0,73
*F. nucleatum*	26.5 ± 0.75	22.0 ± 0.00	24.5 ± 0.25	11.0 ± 0.00	18.5 ± 1.25	-	20.0 ± 0.00
*A.actinomycetemcomitans*	11.6 ± 0.54	12.6 ± 0.22	11.6 ± 0.33	9.30 ± 0.33	13.0 ± 0.00	-	12.3 ± 0,52
*C. albicans*	20.5 ± 0.23	20.5 ± 0.23	21.3 ± 0.33	9,50 ± 0.55	16.3 ± 0.52	17.0 ± 0.00	-
*C. dubliniensis*	24.6 ± 0.37	23.3 ± 0,82	23.3 ± 0.55	14.0 ± 0.00	16.6 ± 0.13	18.0 ± 0.00	-
*C. lusitaniae*	20.3 ± 0.25	20.3 ± 0.25	20.3 ± 0.31	13.0 ± 0.00	16.0 ± 0.00	19.0 ± 0.00	-

*Abbreviations*: *PCV* propolis-based chitosan varnish, *CHV* chitosan varnish, *EPE* ethanol propolis extract, *CHL* chlorhexidine 0.12%, *NYS* nystatin.

Inhibition zones Media and Standard Deviation (M ± SD) of three experiments.

**Table 5:** **Antimicrobial susceptibility test**

Microorganisms	Antimicrobial susceptibility test							
	MIC (μg/mL)				MBC (μg/mL)			
	PCV 15%	CHV	EPE	CHX	PCV 15%	CHV	EPE	CHX
*S. mutans*	4,687	25.4	15.3	17.8	9,375	25.4	15.3	17.8
*S. sanguinis*	5,850	25.4	15.3	17.8	11,710	25.4	15.3	17.8
*S. salivarius*	4,687	25.4	15.3	17.8	4,687	25.4	15.3	17.8
*L. casei*	4,687	25.4	15.3	17.8	4,687	25.4	15.3	17.8

*Abbreviations*: *PCV* propolis-based chitosan varnish, *CHV* chitosan varnish, *EPE* ethanol propolis extract, *CHX* chlorhexidine 0.12%.

Minimum Inhibitory Concentration (MIC) and Minimum Bactericidal Concentration (MBC) of propolis-based chitosan varnish against oral cariogenic bacteria. Planktonic Microtitulation plates test.

### Drug release

**Figure 3:**
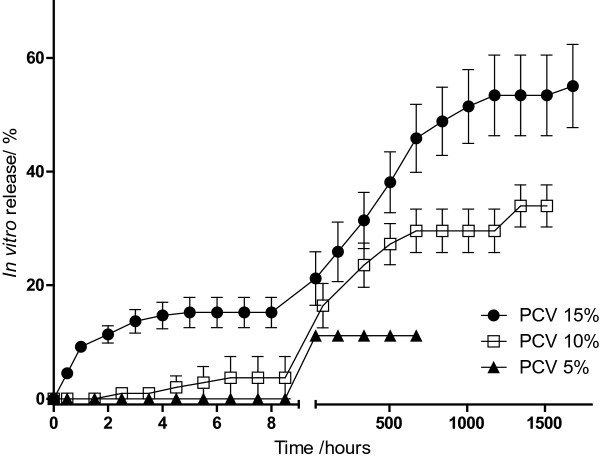
**Sustained release profile of propolis varnishes (PCV) containing 15%, 10% and 5% of propolis.**

All of the varnish formulations have released propolis for more than 24 hours (Figure 3). PCV 5% presented the worst performance, releasing less than 20% of propolis in 24 hours. Since after 4 weeks (672 hours) no more propolis was released, this test was interrupted before de others. PCV 10% had an intermediate performance, releasing more than 30% of the extract in 9 weeks (1512 hours), although only 10% of propolis was released in the first 24 hours. The best sustained release profile was obtained from PCV 15%, which released about 20% of the extract in the first 24 hours. Propolis active components were released for more than one week.

## Discussion

Since dental caries is a disease caused by multiple factors, prevention, based on common risk factors plays an important role on caries management [43, 44]. It can be conducted, for example, by the removal of the microbial deposits, oral hygiene procedures, dietary advice to influence the metabolic activity of the plaque and use of anti-plaque chemical agents such as fluoride toothpaste, gel and varnish [45, 46].

Fluoride is the most important chemical agent used on dental caries prevention [47] and the various topical fluoride interventions have been supporting, over six decades of experimental research, their value as anti-caries measures [46]. But, the high prevalence of dental fluorosis caused concern about his use [48]. Also, there is still a fraction of the population in which the conventional Fluoride regimens seems to have little or no impact on caries prevalence [49]. Moreover, an increased resistance to caries from fluoride has been reported [50].

Antimicrobial therapy is, also, a well-based approach on dental caries prevention [51]. Chlorhexidine, a substance with antimicrobial activity against cariogenic microbiota, has been tried on dental care prevention but, due to the current lack of evidence on long-term clinical outcomes and reported side effects (e.g. yellow-brown staining of the teeth with a mouth rinse, an altered taste sensation, burning sensations of the oral soft tissues, soreness and dryness of the oral tissues, and desquamative lesions and ulcerations of the gingival mucosa), chlorhexidine rinse should not be recommended for caries prevention. Clinical evidence on gels and varnishes containing this substance is also inconclusive [52].

Propolis is a substance which is effective against cariogenic microbiota [13, 53]. But, until now, few formulations containing propolis extract are used on dentistry. Antimicrobial activity of Propolis is associated with flavonoids quercetin, kaemphterol, galangin and pinocembrin and with fenolic acids esters [26, 30, 31]. Anticaries effect of propolis is related to two different mechanisms, such as antimicrobial activity against *Streptococcus mutans* and inhibition of bacterial adherence by inhibition of glucosyltransferase enzymes (GTFs) activity [13, 20], which are considered major properties in the establishment of the cariogenic process [13].

Studies have also shown that chitosan can inhibit *Streptococcus mutans* during the adhesion phase and at successive stages of accumulation displayed a significant antibacterial and plaque reducing action [36, 37], so that chitosan effectively inhibit the initial adherence of oral bacteria onto human tooth surface [38]. Finally, *in vitro* studies have shown that chitosan interferes in the process of the tooth enamel demineralization inhibiting the release of mineral elements [39]. Due its interesting properties, chitosan have been widely used in various long lasting oral formulations [14, 40–42] and exhibited synergistic antiplaque effect when it was associated with chlorhexidine [43]. Table 1 shows the main chemical markers of Brazilian green propolis used in this study. Table 4 shows the averages and standard deviations of the zones of inhibition patterns of chitosan coatings and propolis. Compared with controls isolated chitosan and ethanolic extract of propolis is observed that there is synergism between propolis and chitosan. Varnishes demonstrated greater effectiveness in inhibition of growth of all microorganisms. In this study probably had a synergistic effect between chitosan and propolis when we observe the antimicrobial tests (Table 4).

So, in this work, we developed three pharmaceutical formulations containing both chitosan and green propolis ethanolic extract. Pharmaceutical formulations commonly available on tooth caries prevention are toothpastes, mouthwashes and gels, but drug activity is significantly reduced by these vehicles. So, long-lasting pharmaceutical formulations like varnishes have been recently developed to lead dental caries prevention drugs. Varnishes are pharmaceutical formulations that can form antimicrobial tooth film and protect the tooth surface of caries [16]. The ability to form film on tooth surface is due the presence of polymer in the composition. In our formulations, chitosan was the film-forming polymer.

ATR-FTIR analyses of the formulations showed that there were no new bands in all PCV spectra, which means that propolis constituents interact (probably due hydrogen bonding), but did not react with chitosan to form a new compound. This result was confirmed by antimicrobial activity in *in vitro* tests, which showed synergism between the two products. Synergy is the interaction of multiple elements in a system to produce an effect different from or greater than the sum of their individual effects [54].Synergism occurs when they are associated with drugs that act independently from one another and thus potentiate the activities [55].

The regular casting time of about thirty minutes after varnish application on tooth surface is clinical desired, since it permits an adequate scattering of the formulation without significant casting. After a uniform layer formation, the solvent casting can be rapidly promoted by compressed air, which is commonly found in dentistry office.

The ability to form film on tooth surface was proven by SEM analysis. Images show the formation of a continuous film, which is in intimate contact with tooth surface, suggesting a strong adherence between film and tooth surface.

The thickness of the films was dependent on propolis concentration. Surface images (Figure 2c) suggest a homogeneous and regular formation of the films on tooth surface.

The *in vitro* antimicrobial activity was demonstrated by agar diffusion test. As shown in the Table 4, all the Propolis films were active against *Streptococcus mutans* and leaded the same activity of chlorhexidine films used as positive control. Although it has been described that Chitosan can inhibit *Streptococcus mutans*[37, 44], this was observed in our study, however the zones of inhibition were lower. The concentration of propolis extract, 5, 10 and 15%, in the film did not affect significantly the in vitro antimicrobial activity of the formulations (see confidence interval), probably due the poor solubility and lower diffusion of propolis constituents on agar [48, 49]. However, important that the propolis maintains its antimicrobial properties even when associated with chitosan and varnish. This study demonstrated that all tested propolis conentracoes similarly inhibited all microorganisms. This result allows to choose the varnish concentration of 5% (5% PCV) as the best product for continued study and thus set the dose for application in the mouth. 5% PCV had decreased product costs, and become the best product accepted by patients due to the taste of propolis which had been weaker this concentration.

As shown by SEM analysis, varnish formulations can penetrate on tooth channels. So, we can propose that, in varnish formulations containing 5% of propolis extract, almost all of the extract have penetrated on tooth channels. Since it is more difficult to deliver the extract that is on the channels, only 20% of the extract was released in 4 weeks. In the other hand, since formulations containing 10 or 15% of propolis have larger amount of propolis, part of the extract was in the channels and part of the extract have formed an external cover on tooth surface. So, in these cases, the extract is sustained released over the weeks, but part of the extract which is in the channels is not released even after 10 weeks.

For clinical applications, part of propolis should be delivered in the first 24 hours, in order to kill bacteria already presented on tooth surface and, then, disrupt cariogenic biofilm. On the other hand, since the external varnish film will be removed after 24 hours by brushing, it is important to keep part of the extract on tooth channels. This extract will prevent further formations of cariogenic biofilm. Thinking on this way, it is possible to say that PCV will be used for clinical application because prevent further cariogenic biofilm formation.

## Conclusions

Sustained-release chitosan-based propolis varnishes were successfully developed and characterized in this study. The tooth surface adherence, the ability to form films very fast, the antimicrobial activity similar to chlorhexidine varnishes and the sustained release profile are all characteristic that turns these formulations, PCV 5%, PCV 10%, and PCV 15%, into products suitable for clinical application on dental caries prevention field. All formulations inhibited all tested microorganisms. Additional studies are being conducted to evaluate the safety and the in vivo activity of the formulations.

## Authors’ information

JRF is a student of the Doctoral Program in Pharmaceutical Sciences, UFMG and has experience in Pharmacy, with an emphasis on Pharmacotechniques and Analytical Chemistry, working mainly in the development of polymeric drug delivery systems and the development of systems for analysis by liquid chromatography with high efficiency. Also operates in the area of Phytochemistry applied to pharmaceutics development.

MPL is a student of the Doctoral Program in Dentistry (Paediatric Dentistry), UFMG, researching the properties of natural products for application in dentistry, such as propolis and chitosan in the prevention of dental caries.

TGR is a student of the Doctoral Program in Pharmaceutical Sciences, UFMG and has experience with natural products, development of analytical methods, pharmaceutical technology and parasitology.

ROC is an associate professor at the Federal University of Minas Gerais and has experience in Pharmacy, with emphasis on Chemistry of Natural Products.

ANM is an associate professor at the Federal University of Minas Gerais and has experience in dentistry, with an emphasis in Clinical Dentistry and Periodontology.

VRS is professor of Oral Pathology and Stomatology at the Department of Clinical Pathology and Surgery, Faculty of Dentistry, UFMG. Has experience in dentistry, with emphasis in Oral Pathology, Stomatology and Microbiology, acting on the following topics: medicinal plants and propolis, oral mucosal lesions, oral microbiology, microbial lesions, inflammatory and neoplastic lesions, development of products and medicines for dental use natural-based products. He is currently President of the Brazilian Society of Apitherapy (SBA). Has been studying, in contribution with other authors, the effects of Brazilian propolis in different tissues.

AAGF is an associate professor at the Federal University of Minas Gerais. Currently working in the field of Pharmaceutical Sciences with emphasis in Pharmacotechnical Development with work in development of drug delivery systems using chemically modified natural polymers and to improve the bioavailability profile of drugs in the therapeutic arsenal available.

## Abbreviations

PCV: Propolis - based chitosan varnish
MIC: Minimum inhibitory concentration
MBC: Minimum bactericidal concentration
EPE: Ethanol propolis extract
CHV: Chitosan base varnish
ATR-FTIR: Attenuated total reflection fourier transform infrared spectroscopic imaging
SEM: Scanning electron microscopy
CFU: Colony forming units
CLSI: Clinical and Laboratory Standards Institute
NYS: nystatin
INCQS: Instituto Nacional de Controle de Qualidade (National Institute of Quality Control).
